# Antibiotic resistance in *Pseudomonas aeruginosa*: mechanisms, diagnostic challenges, and omics-based diagnostic solutions

**DOI:** 10.3389/fmicb.2026.1791577

**Published:** 2026-07-30

**Authors:** Md Siddiqur Rahman, Catherine A. Wakeman

**Affiliations:** Department of Biological Sciences, Texas Tech University, Lubbock, TX, United States

**Keywords:** antimicrobial resistance (AMR), diagnostics, machine learning (ML), microfluidic lab on chip, multi omics integration, multidrug resistant (MDR), precision therapeutics, *Pseudomonas aeruginosa*

## Abstract

*Pseudomonas aeruginosa* is considered a multidrug resistant opportunistic pathogen associated with severe infections in immunocompromised patients. Owing to its diverse intrinsic and adaptive resistance strategies, as well as its capacity for horizontal gene transfer, *P. aeruginosa* represents a major contributor to the global antimicrobial resistance burden. Its remarkable ability to evade antibiotics arises from a wide range of mechanisms, including efflux pumps overexpression, porin modification, enzymatic inactivation and target site modifications, alongside phenotypic adaptations such as biofilm and persister cell formation. These complex resistance strategies of the organism have fueled the global emergence of multidrug resistant, extensively drug resistant and even pan drug resistant strains. These strains significantly complicate the treatment strategies. Conventional culture-based diagnostics are still considered the gold standard, yet their delays and limitations in detecting heteroresistance and biofilm-associated tolerance hinder timely therapeutic intervention. Recent advances in omics-based approaches, including genomics, epigenomics, transcriptomics, proteomics, lipidomics, metabolomics, and phenomics, provide powerful alternatives for rapid and precise identification of resistant *P. aeruginosa*. In parallel, innovative diagnostic platforms such as microfluidic lab on chip systems and machine learning driven artificial intelligence further enhance diagnostic resolution. Therefore, multi omics integration, coupled with advanced platforms would be a revolutionary strategy to deliver comprehensive and rapid resistance profiling in precision diagnostics. To convert this potential into practice, proper planning, standardized protocols, clinical validation and cost-effective implementation are urgently needed. Together, these advancements pave the way toward outpacing resistance in *P. aeruginosa* and reducing the global burden of antimicrobial resistance.

## 1 Introduction

*P. aeruginosa* is a rod-shaped gram-negative bacterium. This facultative anaerobe causes a wide array of community-acquired infections (Streeter and Katouli, 2016; Urgancı et al., 2022). It is associated with severe infections primarily among immunocompromised patients. Individuals with chronic obstructive pulmonary disease (COPD), cystic fibrosis (CF), sepsis, cancer, trauma, pneumonia and burns are potentially vulnerable to this infection (Hauser and Rello, 2012; Garcia-Clemente et al., 2020; Qin et al., 2022). Of particular concern, this bacterium is found to be the second leading cause of ventilator associated pneumonia and the third in catheter linked urinary tract infections (UTI) (Cole et al., 2014; Kollef et al., 2014). Additionally, it is also one of the top five pathogens responsible for surgical site infections and comes in tenth among bloodstream infections (Lodise, 2016). Due to its high resistance to antibiotics, the infections caused by *P. aeruginosa* among the hospitalized patients, especially in intensive care units (ICU) are becoming increasingly difficult to treat (Elfadadny et al., 2024). According to the World Health Organization (WHO), *P. aeruginosa* is considered as one of the strongest threats to human health in terms of antibiotic resistance and urgently need novel antibiotics for effective treatment (Spagnolo et al., 2021).

Due to its natural and adaptive resistance capability against antibiotics, *P. aeruginosa* is regarded as multidrug resistant (MDR) pathogen (Qin et al., 2022; Pina-Sánchez et al., 2023). Alongside, certain strains of this bacterium are denoted as high-risk clones and are found worldwide (del Barrio-Tofiño et al., 2020). This resistance against antibiotics is acquired by *P. aeruginosa* in various ways including low outer membrane permeability, chromosomally encoded β-lactamases, mutations, horizontal gene transfer and many more (Torrens et al., 2019; Lorusso et al., 2022; Amisano et al., 2025). In addition, adaptive strategies, such as, biofilm formation, emergence of persister cells etc., bolster its defense against antibiotics (Yan and Bassler, 2019; Haidar et al., 2024). As resistance escalates, conventional diagnostic processes and antibiotics are becoming increasingly ineffective in the treatment of this bacterial infection.

Current diagnostic strategies for *P. aeruginosa* infections face considerable limitations in terms of turnaround time, sensitivity and accurate detection of complex resistance phenotypes (Clarindo Lopes et al., 2024; Hassall et al., 2024). As a result, treatment procedure with appropriate antibiotic therapy is often delayed, since conventional culture-based detection methods can take up to 72 h to identify the bacteria (Tramper-Stranders et al., 2005; Salam et al., 2023; Clarindo Lopes et al., 2024). Moreover, low level resistance in biofilm forming or slow growing populations often remains undetected in standard antibiotic susceptibility testing (AST) procedure (Drevinek et al., 2022). Therefore, the continuously growing resistance in *P. aeruginosa* strains requires more refined diagnostic approaches.

Advanced molecular diagnostics through omics-based diagnostic strategies can be a transformative solution in identifying *P. aeruginosa* in bacterial infections (Babu and Snyder, 2023; Dutta et al., 2024). Incorporating omics technologies into clinical workflows integrated with microfluidic platforms and machine learning (ML) models could potentially improve patient outcomes, even in severe or fatal *P. aeruginosa* infections (Khaledi et al., 2020). As these tools become more accessible, they are expected to redefine diagnostic procedures and treatment strategies for this highly adaptable pathogen.

Antimicrobial resistance in *P. aeruginosa*, its prevalence and emerging technologies to detect this pathogen have been reviewed across several articles in recent years, yet the literature remains fragmented across isolated domains. Existing reviews have addressed resistance mechanisms and antibiogram interpretation (Pang et al., 2019; Fernández-Billón et al., 2023; Giovagnorio et al., 2023; Oliver et al., 2024), global and regional prevalence of MDR/XDR strains (Li et al., 2024; Ramatla et al., 2025; Salleh et al., 2025), therapeutic strategies including novel antibiotics, combination therapy, phage therapy and antimicrobial peptides (Qin et al., 2022; Wood et al., 2023; Elfadadny et al., 2024; Schwartz et al., 2024; Ju et al., 2025; Karampatakis et al., 2025), some omics based AMR platforms (Janiszewska et al., 2022; Fernández-Billón et al., 2023; Clarindo Lopes et al., 2024), microfluidic AST platform engineering (Postek et al., 2022; Hattab et al., 2024; Reszetnik et al., 2024; Wu and Mu, 2024), and AI/ML-driven AMR prediction (Sakagianni et al., 2023; de la Lastra et al., 2024; Olatunji et al., 2024; Bilal et al., 2025; Zhang et al., 2025). However, no existing review systematically maps each *P. aeruginosa* resistance mechanism to the omics platform best suited to detect it and integrates all omics layers within a unified framework coupled with microfluidics and ML to address the clinical translation roadmap.

The present review fills this gap. To our knowledge, it is the first comprehensive review integrating the multi-omics to microfluidic lab-on-chip platforms and ML-driven analytics as a unified diagnostic framework for antibiotic-resistant *P. aeruginosa*. This review also describes all the known resistance mechanism in *P. aeruginosa* and illustrates how each omics layer resolves distinct resistance strategies. Finally, it provides a critical discussion of clinical, regulatory, cost and implementation barriers currently preventing bedside translation and a forward-looking framework for rapid resistance profiling to guide precision antimicrobial therapy.

## 2 Antibiotic resistance genes and mechanisms

*P. aeruginosa* exploits a number of intrinsic and acquired resistance mechanisms when exposed to antibiotics (Qin et al., 2022; Pina-Sánchez et al., 2023). The known genetic mechanisms and phenotypic alterations that are responsible for antibiotic resistance in this bacterium are highlighted below, and are summarized in Table 1.

**TABLE 1:** Summary of major antibiotic resistance mechanisms in *P. aeruginosa* including key genes, functional features and affected antibiotics.

No	Resistance mechanism	Key genes/regulators	Functional description	Representative antibiotics affected
1	Efflux pump overexpression	*mexAB-oprM, mexXY-oprM, mexCD-oprJ, mexEF- oprN; regulators: mexR, nalC, nalD, mexZ, mexT, mexS*	Active efflux of multiple antibiotics; repressor gene mutations cause overexpression.	P-lactams, fluoroquinolones, tetracyclines, macrolides, aminoglycosides, chloramphenicol
2	Porin modifications /membrane alterations	*oprD, opdP, oprF, pmrA/pmrB, phoPQ*	Mutations or deletions in porins limit drug influx; lipid A modification decreases antibiotic binding.	Carbapenems (imipenem, meropenem), polymyxins
3	Enzymatic inactivation of antibiotics	ß-lactamases: *ampC, blaVIM, blaIMP, blaNDM’*, AMEs: *aac(6)-Ib, ant(2”)-Ia, aph(3’)-IIb; others: catB7, fosA, crpP, ereA, mphA*	Enzymatic hydrolysis or chemical modification of antibiotics.	P-lactams, aminoglycosides, macrolides, fosfomycin, chloramphenicol, fluoroquinolones
4	Target site modification	*gyrA, parC, rpoB, folP, ftsl, rmtA, rmtB, armA*	Mutations alter target binding sites	Fluoroquinolones, aminoglycosides, rifampicin, sulfonamides, P-lactams, polymyxins
5	Phenotypic adaptations	*lasR, rhlR, pel, psi, algD, ndvB, rnticA*	Biofilm and persister formation reduce drug penetration and metabolic activity.	All antibiotic classes

### 2.1 Efflux pumps

In *P. aeruginosa*, efflux pumps expel toxic compounds, including antibiotics, and play a key role in multidrug resistance (Lorusso et al., 2022). Among these pumps, the chromosomally encoded Resistance Nodulation Division (RND) family are the most critical for antibiotic resistance (Avakh et al., 2023). While *P. aeruginosa* harbors approximately 12 RND efflux pumps, MexAB-OprM, MexXY-OprM, MexCD-OprJ, and MexEF-OprN are clinically the most significant (Avakh et al., 2023). Each of these pumps functions as tripartite protein complexes consisting of an inner membrane transporter protein, a periplasmic adapter protein and an outer membrane channel protein (Lorusso et al., 2022). However, all of these pumps do not expel the same antibiotics, rather, they exhibit overlapping but distinct substrate specificities (Masuda et al., 2000; Okamoto et al., 2002).

Among the RND pumps, MexAB-OprM can expel β-lactams, fluoroquinolones, tetracyclines, macrolides and chloramphenicol (Masuda et al., 2000; Li and Plésiat, 2016). The overexpression of this pump is driven by mutations in the negative regulators mexR, nalC, or nalD (Aguilar-Rodea et al., 2022). Another efflux pump, MexXY-OprM is inducible by ribosome-targeting antibiotics and is the only RND pump in *P. aeruginosa* that exports aminoglycosides, in addition to macrolides, tetracyclines and quinolones (Masuda et al., 2000; Kavanaugh et al., 2025). Its key repressor MexZ is relieved by the anti-repressor ArmZ upon antibiotic exposure (Kawalek et al., 2019). MexCD-OprJ is another RND pump which is overexpressed through *nfxB* mutations (Fraud et al., 2008; Monti et al., 2013). It confers resistance to a subset of β-lactams (piperacillin, cefepime, meropenem), fluoroquinolones and macrolides (Fraud et al., 2008). MexEF-OprN is similarly activated through the MexT/MexS regulatory axis, and upon activation, it confers resistance to chloramphenicol, fluoroquinolones, and trimethoprim (Kumar and Schweizer, 2011; Avakh et al., 2023).

Along with RND pumps, *P. aeruginosa* has other efflux families including Major Facilitator Superfamily (MFS), ATP-Binding Cassette (ABC) transporters, Small Multidrug Resistance (SMR) family and Multidrug and Toxic compound Extrusion (MATE) family (Piddock, 2006; Sun et al., 2014; Ahmad et al., 2024). Overexpression of MFS pump genes, *mfs1* and *mfs2*, can increase resistance to aminoglycosides and quinolones (Dulyayangkul et al., 2021). Additionally, ABC transport systems can confer resistance to tobramycin or norfloxacin (Zhang and Mah, 2008; Poudyal and Sauer, 2018). Recent study also reveals that SMR efflux transporter (MdtJI) protects *P. aeruginosa* against polycationic antibiotics by actively pumping these antibiotics out of the cell (Adamiak et al., 2024). Finally, the MATE family efflux pump, PmpM is found to confer resistance in *P. aeruginosa* to fluoroquinolones (He et al., 2004).

Collectively, efflux pump upregulation is the major cause and can be considered the first step in the acquisition of resistance to antibiotics found in *P. aeruginosa* (Schmalstieg et al., 2012). These pumps are responsible for reducing the intracellular concentration of the antibiotic to a sub-inhibitory concentration (Fernández and Hancock, 2012; Huang et al., 2022).

### 2.2 Porin modifications and membrane alterations

The outer membrane of *P. aeruginosa* is naturally less permeable to external compounds, including antibiotics (Angus et al., 1982; Chevalier et al., 2017). This permeability barrier develops from several factors. The absence of large porins like OmpF and OmpC is one of them (Chevalier et al., 2017; Amisano et al., 2025). In addition, the outer membrane of *P. aeruginosa* is composed of highly anionic lipopolysaccharide (LPS) molecules, which reduce the uptake of many hydrophilic antibiotic molecules (Manrique et al., 2024).

One of the most important porins through which antibiotics, particularly carbapenems enter into *P. aeruginosa* is *OprD* (Tamber et al., 2006; Li et al., 2012). Mutations, promoter deletions or insertion of transposable elements like IS256 in *oprD* gene can lead to the emergence of carbapenem resistant only *P. aeruginosa* (CROPA) strains (Ochs et al., 1999; Wolter et al., 2004; Wang et al., 2025). Based on recent findings, *oprD* mutations are considered the most common cause of carbapenem resistance in *P. aeruginosa* (Livermore, 2002). Additionally, mutations or deletions in another porin gene *opdP*, can cause increased resistance to carbapenems in *P. aeruginosa*, especially when *oprD* gene is already mutated or absent (Amisano et al., 2025). Some other outer membrane channels, such as OprF and OpdK may also play a role in controlling antibiotic uptake (Bouffartigues et al., 2012; Eren et al., 2013).

In addition to porin changes, *P. aeruginosa* can undergo structural modifications of its outer membrane lipopolysaccharide (LPS), further reducing antibiotic influx (Huszczynski et al., 2019; Sabnis and Edwards, 2023). These modifications, including the addition of cationic groups to lipid A through the pmrA/pmrB two-component system, are particularly relevant to polymyxin resistance (Ayoub Moubareck, 2020).

Overall, porin modifications and membrane alterations in *P. aeruginosa* are crucial mechanisms to develop potential barrier to antibiotic influx. Additionally, mutations can further increase the resistance by downregulating or eliminating specific porins and by overactivating lipid A modifying genes.

### 2.3 Enzymatic inactivation of antibiotics

Enzymatic inactivation of drugs is a critical adaptive resistance mechanism employed by *P. aeruginosa*. This strategy generally involves the production of β-lactamases, aminoglycoside-modifying enzymes and other drug-inactivating enzymes targeting various antibiotic classes (Torrens et al., 2019; Thacharodi and Lamont, 2022).

#### 2.3.1 β-lactamases

Upon exposure to β-lactam antibiotics, *P. aeruginosa* produces β-lactamase enzymes that hydrolyze the β-lactam ring of the antibiotics, such as penicillins and cephalosporins (Petri, 2006; Glen and Lamont, 2021). Interestingly, genes that encode these enzymes can be either chromosomal or acquired through horizontal gene transfer (HGT) (Torrens et al., 2019; Hussain et al., 2021).

The chromosomally encoded AmpC β-lactamase is a major contributor to intrinsic resistance (Torrens et al., 2019). Its expression is regulated by AmpR (activator) and AmpD (repressor) (Jones et al., 1997). Generally, in a wild type *P. aeruginosa*, AmpC levels are low, as AmpR is repressed by UDP-MurNAc-pentapeptide binding (Klycheva et al., 2025). However, β-lactam induced cell wall disruption generates muropeptides that displace this precursor, and thus AmpR is activated and triggers AmpC overproduction (Vadlamani et al., 2015; Tamma et al., 2019). Moreover, mutations in *ampD* prevent muropeptide degradation (Schmidtke and Hanson, 2006). This confers resistance to expanded-spectrum cephalosporins and penicillins (Pérez-Gallego et al., 2016). Additionally, mutation in the AmpC Ω-loop is found to reduce susceptibility to newer combinations such as ceftolozane-tazobactam by diminishing tazobactam binding affinity and enhancing ceftolozane hydrolysis (Skoglund et al., 2018; Chen et al., 2024).

Through HGT, *P. aeruginosa* can also acquire transferable β-lactamases, including extended-spectrum β-lactamases (ESBLs) and metallo-β-lactamases (MBLs) (Glen and Lamont, 2021; Halat and Moubareck, 2022). While ESBLs confer resistance to penicillins, most cephalosporins, and aztreonam, MBLs hydrolyze nearly all β-lactams including carbapenems (Queenan and Bush, 2007; Halat and Moubareck, 2022).

#### 2.3.2 Aminoglycoside-modifying enzymes

Aminoglycoside-modifying enzymes (AMEs) are among the most widespread mechanisms by which *P. aeruginosa* develops resistance to antibiotics (Ahmadian et al., 2021; Thacharodi and Lamont, 2022). So far, researchers have identified three types of aminoglycoside-modifying enzymes in *P. aeruginosa*. These include acetyltransferases (AACs), nucleotidyltransferases (ANTs) and phosphotransferases (APHs) (Serio et al., 2017; Thacharodi and Lamont, 2022).

Several AME genes have been identified in clinical isolates of *P. aeruginosa*, including *aac(6’)-Ib, aac(3)-II, aph(3’)-VI, aph(3’)-IIb, aadA1*, and *ant(2”)-Ia* (Ahmadian et al., 2021; Thacharodi and Lamont, 2022). Among them, the enzyme AAC(6’)-Ib, one of the most prevalent, confers resistance to amikacin and tobramycin by acetylating the 6’-amino group of aminoglycosides (Reeves et al., 2020; Thacharodi and Lamont, 2022). Similarly, APH(3’)-IIb phosphorylates the 3’-hydroxyl group of gentamicin and tobramycin, effectively inactivating these antibiotics (Thacharodi and Lamont, 2022). In addition, the *aadA* family genes encode enzymes that adenylate streptomycin and spectinomycin, and thus blocks their antimicrobial activity (Stern et al., 2018). These modifications reduce the ability of antibiotics to interact with ribosomal targets, and thus prevent the inhibition of protein synthesis by the antibiotics.

#### 2.3.3 Other drug inactivating enzymes

Beyond β-lactamases and AMEs, *P. aeruginosa* harbors additional drug-inactivating enzymes that target other antibiotic classes. These include chloramphenicol acetyltransferase (*catB7*), fosfomycin-inactivating glutathione S-transferase (*fosA*), and the recently described ciprofloxacin-modifying kinase (*crpP*) genes (Wang and Liu, 2004; Chávez-Jacobo et al., 2018; Pipitone et al., 2023). In addition, macrolide resistance is mediated by erythromycin esterases (*ereA*) and macrolide phosphotransferases (*mphA*, *mphB*), which enzymatically inactivate erythromycin and azithromycin (Zieliński et al., 2021). While individually these enzymes may contribute modestly to resistance, their co-occurrence with efflux pumps and β-lactamases in MDR isolates can compound therapeutic failure.

### 2.4 Target site modifications

In *P. aeruginosa*, target site modification involves mutations that alter the structure of the antibiotic target and prevent the antibiotic from binding and exerting its effect (Lambert, 2005). Point mutations are particularly common in *P. aeruginosa* under antibiotic selective pressure (Alonso et al., 1999; Feng et al., 2025).

Fluoroquinolones (FQs), such as ciprofloxacin and levofloxacin, target DNA gyrase and topoisomerase in *P. aeruginosa* (Hooper and Jacoby, 2016). Resistance against these antibiotics is predominantly driven by mutations in the target genes *gyrA* and *parC* (Ng et al., 1996; Dasgupta et al., 2018). The most common mutations at T83I in GyrA and S87L in ParC alter the fluoroquinolone binding site. Similarly, resistance to the other classes of antibiotics, such as rifampicin and sulfonamide, arises from mutations in the RNA polymerase β-subunit gene (*rpoB*) and dihydropteroate synthase gene (*folP*), respectively (Sköld, 2000; Cai et al., 2017; Venkatesan et al., 2022).

Polymyxins, such as colistin and polymyxin B, target the lipid A component of the outer membrane lipopolysaccharide (LPS) in *P. aeruginosa*, disrupting membrane integrity (Ayoub Moubareck, 2020). In polymyxin resistance, mutations in the two-component regulatory genes *pmrA*/*pmrB* lead to the addition of 4-amino-4-deoxy-L-arabinose to the phosphate groups of lipid A (Moskowitz et al., 2004; Miller et al., 2011; Chin et al., 2015). This modification reduces the net negative charge of lipid A and consequently decreases polymyxin binding affinity.

Another clinically significant target modification is in Penicillin-Binding Protein 3 (PBP3), which is involved in the final stages of peptidoglycan synthesis of cell wall formation. Mutations or insertions in the *ftsI* gene, can alter the structure of PBP3 (Glen and Lamont, 2024). This leads to reduced affinity of antibiotics like ceftazidime and penicillin for PBP3. *P. aeruginosa* isolates from cystic fibrosis patients generally exhibit this resistance mechanism.

Another important target site modification is mediated by 16S rRNA methyltransferases genes like *rmtA*, *rmtB*, *rmtC/F*, or *armA* (Doi and Arakawa, 2007; Mohanam and Menon, 2017). The enzymes add a methyl group to specific nucleotides within the 16S rRNA, causing a steric hindrance for aminoglycosides to their ribosomal target site binding (Mohanam and Menon, 2017; Srinivas et al., 2023). This modification effectively renders the bacteria highly resistant to most clinically useful aminoglycosides.

Overall, target site mutations are potential contributors to antibiotic resistance in *P. aeruginosa*, especially for fluoroquinolones and aminoglycosides.

### 2.5 Phenotypic adaptations

Beyond specific genes, *P. aeruginosa* can undergo phenotypic adaptations that make it transiently more resistant or tolerant to antibiotics (Moradali et al., 2017; Ciofu and Tolker-Nielsen, 2019). The best-known examples are biofilm development and persister cell formation (Lewis, 2008).

In biofilms, *P. aeruginosa* cells are embedded in a self-produced exopolysaccharide (EPS) matrix, which is basically composed of alginate, Pel and Psl (Gheorghita et al., 2023; da Cruz Nizer et al., 2024). Pel is a glucose-based polymer, which is important for cell-to-cell adhesion and the biofilm stabilization (Jennings et al., 2015). On the other hand, Psl is a mannose-rich polymer, critical for initial surface attachment and immune evasion (Ma et al., 2007). In addition, by forming a diffusion barrier, the EPS slows down antibiotic penetration and creates gradients of nutrients and oxygen. Biofilm-grown bacteria can withstand antibiotic concentrations 10–1,000 fold higher than genetically equivalent planktonic cells (Thöming and Häussler, 2022). Chronic *P. aeruginosa* infections, such as those in cystic fibrosis airways, are often associated with biofilm formation, making them recalcitrant to therapy (Hassett et al., 2010; Borisova et al., 2025).

Within biofilms, bacterial cells can enter a slow-growing or dormant state, known as persister cells (Wood et al., 2013). These cells are highly tolerant to antibiotics, since the drugs cannot exert their effects due to the reduced metabolic activity of the bacteria. Mutations in regulatory genes, such as *ndvB*, which is involved in the synthesis of periplasmic cyclic glucans, can promote biofilm formation and intrinsic resistance in *P. aeruginosa* (Hall et al., 2018). Biofilm mediated tolerance is not caused by a single gene. Instead, it is associated with global regulators, including LasR/RhlR quorum sensing systems and cyclic-di-GMP regulators (Hall et al., 2018; Chu and Yang, 2024). Furthermore, the biofilm environment promotes emergence of mutated strains over time in chronic *P. aeruginosa* infections, such as, those in chronic cystic fibrosis, and facilitates HGT of resistance plasmids (Borisova et al., 2025). Another phenotypic adaptation is mucoid formation by *P. aeruginosa*. In this scenario, the bacteria overproduce alginate, often due to *mucA* gene mutations (Colbert et al., 2018). This mucoid phenotype is common in CF isolates and can shield bacteria from antibiotics and host immune response.

In summary, *P. aeruginosa* utilizes multifaceted resistance strategies against antibiotics. The convergence of these diverse resistance strategies within single isolates has driven a global escalation in resistant *P. aeruginosa* strains, which is discussed in Section 3. Understanding which resistance mechanisms are active in a given isolate is therefore essential for effective treatment. However, as discussed in Section 4, conventional diagnostics struggle to resolve this complexity in real time. Omics-based approaches (Section 5) can address these challenges.

## 3 Prevalence of resistant strains

According to CDC and ECDC, antibiotic resistance is categorized as MDR (resistant to ≥1 agent in ≥3 classes), XDR (susceptible to ≤2 classes) or PDR (resistant to all agents in all antibiotic classes) (Magiorakos et al., 2012; Eatemadi et al., 2021). The continuous rise of these antibiotic resistant *P. aeruginosa* isolates poses a serious global health threat. The 2019 Global Burden of Bacterial AMR (GBD) estimated 4.95 million deaths associated with bacterial AMR and 1.27 million deaths attributable worldwide (Murray et al., 2022; Ranjbar and Alam, 2024). *P. aeruginosa* was identified as one of the top six pathogens that accounted for 3.57 million AMR-associated deaths and 929,000 AMR-attributable deaths (Murray et al., 2022). Updated GBD modeling for 1990–2021 estimated 4.71 million AMR-associated deaths and 1.14 million AMR-attributable deaths in 2021 (Naghavi et al., 2024). In addition, forecasts suggest 1.91 million deaths attributable to AMR and 8.72 million associated deaths in 2050, which is approximately 70% increase in deaths vs. 2022 (Naghavi et al., 2024).

Surveillance studies and meta-analyses indicate alarmingly high rates of MDR and XDR strains in healthcare settings worldwide (Schwartz et al., 2024). MDR/XDR rates in *P. aeruginosa* strains is estimated 15–30% in several regions and higher in some large hospital cohorts. A meta-analysis of 163 studies published between 2014 and 2024 estimated that 34.7% of *P. aeruginosa* isolates were resistant to carbapenems (Ramatla et al., 2025). This is particularly concerning, as carbapenem-resistant strains are typically resistant to multiple antibiotic classes. Notably, the 2024 WHO Bacterial Priority Pathogens List classified carbapenem-resistant *P. aeruginosa* in the high priority tier, since this pathogen is highly transmissible in healthcare settings and carries substantial mortality (World Health Organization, 2024).

According to the International Network for Optimal Resistance Monitoring (INFORM) database, the global rate of MDR *P. aeruginosa* is between 11.5 and 24.7% (Sader et al., 2018). A 2023 meta-analysis of ventilator-associated pneumonia (VAP) found that MDR *P. aeruginosa* accounted for 33% of cases worldwide (Li et al., 2024). In European hospitals, the rates of MDR and XDR *P. aeruginosa* strains range from 15 to 30%, with up to 47.6% carbapenem resistance (Ramatla et al., 2025). In contrast, VAP-associated MDR prevalence is reported at about 19.7% in the United States (Li et al., 2024). Recent data from Asia and Africa estimate that 46% of *P. aeruginosa* strains are MDR and nearly 20% are XDR (Salleh et al., 2025). PDR *P. aeruginosa* remains uncommon, but a scoping review reported a prevalence of 15.2%, predominantly from hospitals in countries such as India and Iran (Kamal et al., 2024).

The burden of resistant *P. aeruginosa* varies considerably across clinical settings. It is considered the fourth most common cause of all healthcare-associated infections (Weiner-Lastinger et al., 2020). *P. aeruginosa* accounts for more than 16% of all nosocomial infections and up to 23% of infections acquired in intensive care units (ICUs) (Schwartz et al., 2024). Among cystic fibrosis (CF) patients, chronic *P. aeruginosa* infection affects 60–70% of adults and is the leading cause of progressive pulmonary deterioration, with the emergence of MDR and mucoid phenotypes over time being almost inevitable (Crull et al., 2018; Acosta et al., 2020; Jarzynka et al., 2023). Burn patients represent another high-risk population and around 55% infections in these patients are caused by *P. aeruginosa* (Bhatt et al., 2015). It has been estimated that at least 50% of all deaths caused by burn infections frequently associated with *P. aeruginosa* (Anvarinejad et al., 2014; Azam et al., 2024). Additionally, evidence supports that MDR *P. aeruginosa* accounts for roughly one-quarter to over one-third of bloodstream infections and is consistently associated with substantially higher mortality, which threatens effective management of sepsis, pneumonia, urinary tract infections and wound infections (Recio et al., 2020; Schwartz et al., 2024).

These figures suggest that MDR, XDR, and PDR strains of *P. aeruginosa* are rising globally, driven by antibiotic misuse and the organism’s ability to frequently acquire resistance genes. Effectively addressing this challenge requires not only surveillance and stewardship but also diagnostic tools capable of rapidly identifying resistant strains and guiding targeted therapy. However, as discussed below, conventional diagnostic methods face significant limitations in meeting this need.

## 4 Current diagnostic challenges

Timely and accurate diagnosis of *P. aeruginosa* infection, along with its antibiotic susceptibility profile, is crucial for effective treatment. However, current conventional diagnostic methods have significant challenges in dealing with this pathogen. To date, traditional culture-based techniques remain the gold standard worldwide, but they are often slow and have limitations that impact patient outcomes and epidemiological control.

### 4.1 Time delays

**FIGURE 1:**
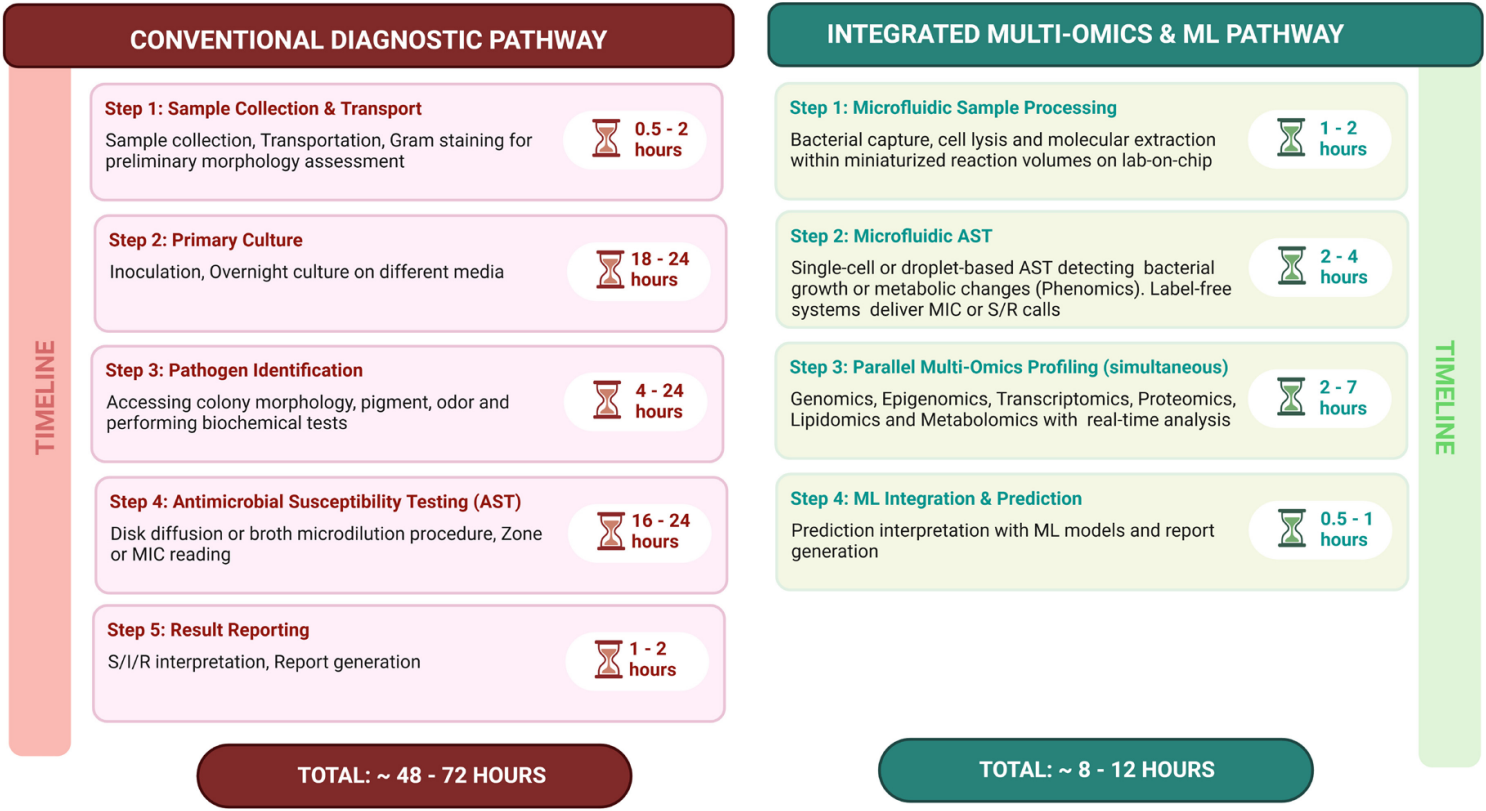
Conventional versus multi-omics ML diagnostic pathway for *P. aeruginosa*. The left panel illustrates the conventional culture-based workflow, with a total turnaround time of 48–72 h and a binary susceptibility output (S/I/R). The right panel depicts the proposed integrated multi-omics ML pathway, in which microfluidic antimicrobial susceptibility testing and parallel multi-omics profiling run concurrently, with ML-driven prediction and automated reporting potentially compressing the workflow within a single clinical workday (∼8–12 h). This compressed turnaround is accompanied by richer clinical outputs, including resistance mechanism identification and biofilm risk stratification. Projected timings for the multi-omics pathway are derived from the reported performance of individual platforms operating independently and do not represent a validated, fully integrated clinical system. Created in https://BioRender.com.

In the case of the conventional diagnostic process, as shown in Figure 1, it can take around 48–72 h from specimen collection to obtain a full identification and AST report for *P. aeruginosa* (Tramper-Stranders et al., 2005; Salam et al., 2023; Wenzler et al., 2023). However, one study reported median times of about 37 h to organism identification and around 60.5 h to AST result using standard methods (MacVane et al., 2020). This delay is problematic in severe infections like sepsis, where each hour of delay in effective therapy increases mortality. *P. aeruginosa* septicemia can progress rapidly, and waiting 2–3 days for AST results means clinicians must rely on broad empiric therapy (Osih et al., 2007; Zakhour et al., 2022). This broad-spectrum antibiotic application may be ineffective if the strain is resistant and can be harmful to the beneficial microbiota (Modi et al., 2014). Therefore, faster diagnostic turnaround is essential for the early initiation of effective medication.

### 4.2 Identification challenges

Identification of *P. aeruginosa* through morphology observation and simple biochemical tests is generally straightforward. However, challenges remain in certain scenarios. For example, in polymicrobial infections, such as, cystic fibrosis sputum or wound infections, *P. aeruginosa* can be overgrown or masked by other organism, making subculturing labor-intensive (Reece et al., 2021). Furthermore, *P. aeruginosa* can adopt small colony variant forms under antibiotic pressure. These colonies may be difficult to recognize or may grow more slowly, resulting in delayed identification (Evans, 2015). In addition, current diagnostics do not readily provide information on virulence traits or quorum-sensing activity in polymicrobial infections.

### 4.3 Susceptibility testing issues

Traditional AST for *P. aeruginosa* usually involves disk diffusion or broth dilution methods. Several diagnostic challenges may arise with these techniques (Livermore et al., 2012; Al-Daghistani et al., 2025).

*P. aeruginosa* can develop heteroresistance to particular antibiotics, meaning that subpopulations with different resistance levels coexist within the same isolate (Evans, 2015; Chen, 2023; Al-Daghistani et al., 2025). In such cases, misinterpretation may occur. For example, carbapenem disk diffusion tests may appear susceptible, while a resistant subpopulation may emerge later (Al-Daghistani et al., 2025). In addition, inducible AmpC mediated resistance represents another challenge. *P. aeruginosa* AmpC can be induced by certain β-lactams. If not tested properly, laboratories may fail to detect strains that appear ceftazidime-susceptible but rapidly become resistant during therapy due to AmpC induction (Livermore et al., 2012).

Furthermore, standard AST is generally performed on planktonic *P. aeruginosa*, but in chronic infections these bacteria can often reside in biofilms. There is currently no standardized clinical AST for biofilm-grown bacteria, though research-based methods exist (Coenye, 2023). This discrepancy means that an antibiotic reported as susceptible based on planktonic MIC may still fail clinically if the infection involves biofilm.

These limitations including the 48–72 h turnaround of culture-based AST, the inability to detect heteroresistance and biofilm-associated tolerance, and the failure to identify underlying resistance mechanisms, collectively highlight the need for a fundamentally different diagnostic paradigm. Omics-based approaches, reviewed in the following section, offer the molecular resolution required to address each of these shortcomings.

## 5 Advanced omics diagnostics

Understanding the super complex nature of antimicrobial resistance in *P. aeruginosa* requires a comprehensive approach that captures genetic, regulatory, metabolic and phenotypic determinants of adaptation. To overcome the shortcomings of conventional methods, omics based diagnostic technologies provide a framework for elucidating these complex resistance mechanisms. This advanced diagnostic platform includes genomics, epigenomics, transcriptomics, proteomics, lipidomics, metabolomics and phenomics. A consolidated comparison of these omics platforms is provided in Table 2. Each of these omics type has unique insights. Below, we review these omics-based technologies in order.

**TABLE 2:** Overview of omics-based diagnostic technologies in *P. aeruginosa* resistance profiling.

Omics category	Genomics	Epigenomics	Transcriptomics	Proteomics	Lipidomics	Metabolomics	Phenomics
Key technology	- WGS	- SMRT sequencing (PacBio)	- RNA sequencing	- LC-MS/MS - MALDI-TOF Biotyper	- LC-MS/MS lipid profiling - MALDI-TOF lipid fingerprinting	- LC-MS/GC-MS metabolite profiling, NMR	- Biolog Microarrays - Accelerate Pheno - Microfluidic AST
Output	SNPs, mobile elements, resistance islands	Methylation maps	Transcript abundances	Protein-level	Outer membrane remodeling and charge modulation	Metabolite profiles	Growth/metabolic response, substrate use, morphology
Identifies resistance type	3, 4	1, 5	1, 5	1, 3	2, 5	3, 5	5
Advantages	- Comprehensive survey of all genetic determinants - Cost-effective and scalable	- Uncovers reversible regulation mechanisms - New insight into adaptive resistance	- Connects genotype to phenotype - Provides functional context	- Suitable for mixed or complex samples - Useful for studying microbial interactions and substrate use	- Highly specific to membrane changes - Directly detects lipid A modifications	- Provides physiological snapshot of microbial ecosystem - Identifies potential metabolic biomarkers	- Detects phenotypic heterogeneity - Most technologies provide rapid and scalable platforms
Disadvantages	- No information on functional properties - Affected by host DNA contamination	- Weak sensitivity in some cases - Complex analysis	- RNA instability - Affected by host RNA contamination	- Low abundance proteins may be missed - High instrumentation cost	- Complex lipid extraction and annotation process - Some lipid classes poorly represented	- Lack of comprehensive reference libraries - Difficult to trace changes back to genes	- Outcomes can vary with conditions - Some systems require high setup cost or expert handling

Resistance types: 1—Efflux pump overexpression. 2—Porin modifications / membrane alterations. 3—Enzymatic inactivation of antibiotics. 4—Target site modification. 5—Phenotypic adaptations.

### 5.1 Genomics

Genomics has emerged as a powerful and comprehensive approach to pathogen identification (Vashisht et al., 2023). At the center of this approach is whole-genome sequencing (WGS), which decodes the complete DNA content of a bacterial isolate, thereby revealing the full repertoire of genetic determinants that contribute to resistance (Oniciuc et al., 2018). By mapping single nucleotide polymorphisms, insertions, deletions, mobile genetic elements and horizontally acquired resistance genes, WGS can provide a detailed resistome profile in a single assay (Oniciuc et al., 2018). For example, WGS readily detects resistance genes involved in enzymatic drug inactivation, such as diverse β-lactamase families and aminoglycoside-modifying enzymes. It also identifies target-site modifications, including mutations in *gyrA* and *parC*. In addition, it is also capable of inferring changes in other resistance categories, such as efflux pump overexpression and membrane alteration through associated mutations. As a result, this method enables prediction of antibiotic susceptibility by comparing genomic data against curated resistance gene databases. This allows clinicians and researchers to infer which antibiotics are likely to fail or succeed against a given isolate. In one study of 494 *P. aeruginosa* isolates, ML models using WGS data accurately predicted resistance to imipenem, meropenem, piperacillin-tazobactam, and levofloxacin (Cao et al., 2024). These findings illustrate the growing reliability of genomic predictions when chromosomal mutations and regulatory variants are thoroughly incorporated into analytical models.

Some advanced tools for identifying antimicrobial resistance genes in bacterial genomes have been developed. Among them ResFinder, AMRFinderPlus, PointFinder etc., are noteworthy (Zankari et al., 2017; Feldgarden et al., 2019; Florensa et al., 2022). Some of these tools can achieve more that 98% consistency between genotypes and phenotypes correlation (Feldgarden et al., 2019). However, some development process is still ongoing in quality control and making them accessible to healthcare.

Clinically, some laboratories in the United Kingdom have already implemented routine WGS for bloodstream isolates (Genome UK, 2022). This approach allows rapid identification of high-risk clones, surveillance of outbreak lineages and early detection of emerging resistance genes. Furthermore, advances in metagenomic sequencing now enable WGS to be conducted directly from patient samples (Cao et al., 2024). This culture-independent approach has demonstrated success in detecting *P. aeruginosa* and its resistance determinants from complex specimens such as sputum.

Despite its transformative potential, genomic diagnostics face several limitations. WGS requires substantial infrastructure, trained personnel and specialized bioinformatics pipelines (Christensen et al., 2015; Osei Sekyere, 2025). Therefore, WGS is more resource-intensive than routine diagnostic assays. In addition, because sequencing captures only the genetic blueprint, it provides no direct information on functional properties such as gene expression, protein activity, or phenotypic adaptation (Brlek et al., 2024). This limitation is particularly relevant for *P. aeruginosa*, where resistance often results from regulatory shifts rather than fixed mutations. Single-gene prediction models may therefore underestimate true resistance levels. In addition, novel or uncharacterized mutations may be absent from current databases such as CARD or ResFinder, which may lead to false-negative predictions. Furthermore, data quality can be affected by factors, such as host DNA contamination during sample preparation (Dohál et al., 2020; Brlek et al., 2024). Finally, interpreting genomic data remains challenging, as *P. aeruginosa* possesses a highly plastic large size genome (∼6.3 Mb) having extensive accessory genes (Karampatakis et al., 2025). Consequently, genotype-to-phenotype prediction accuracy for *P. aeruginosa* remains lower (up to 85% for most antibiotics) than for less genomically complex species (Madden et al., 2024). This underscores the need for complementary omics layers to improve diagnostic reliability.

Overall, while not sufficient as a standalone predictor of phenotype, WGS forms an essential foundation for integrated, multi-omics approaches and continues to shape the future of precision diagnostics.

### 5.2 Epigenomics

Epigenomic analysis represents an emerging frontier in antimicrobial resistance research. This method provides insights into regulatory layers that extend beyond static DNA sequence variation (Gomase and Tagore, 2008). In *P. aeruginosa*, DNA methylation, primarily N6-methyladenine and to a lesser extent, 5-methylcytosine is increasingly recognized as a contributor to gene regulation, virulence and stress adaptation (Doberenz et al., 2017). However, modern long-read sequencing technologies have evolved to detect these modifications. Among them, PacBio Single-Molecule Real-Time (SMRT) sequencing and Oxford Nanopore sequencing are noteworthy. They can simultaneously determine nucleotide sequence and detect methylation signatures in native DNA without chemical conversion (Flusberg et al., 2010; Barros-Silva et al., 2018; Liu and Conesa, 2025). These platforms have enabled genome-wide bacterial methylome mapping. Thus, they provide new opportunities to investigate how epigenetic modifications influence resistance phenotypes.

Although epigenomics in *P. aeruginosa* remains understudied, available evidence suggests that methylation may fine-tune expression of genes associated with antibiotic tolerance, efflux mechanisms and membrane remodeling (Ghosh et al., 2020; Nass and Zaher, 2025). Dynamic switching of methylation states at promoter regions can alter transcriptional output of efflux pumps or porins. Additionally, studies have identified methyltransferase motifs and orphan methylases in *P. aeruginosa*, suggesting methylation patterns may contribute to patho-adaptation (Doberenz et al., 2017). Therefore, methylation profiling may reveal regulatory architectures, that can control biofilm formation, LPS modification pathways or metabolic circuits.

Despite its promise, epigenomics faces substantial limitations in antibiotic resistance identification. Functional interpretation of many methylation marks remains challenging, since lots of detected sites lack experimentally validated regulatory roles and correlations with gene expression (Heyn and Esteller, 2012). Consequently, epigenomic insights are still largely exploratory but hold significant potential for uncovering novel regulatory layers involved in antibiotic resistance and tolerance.

### 5.3 Transcriptomics

While WGS elucidates the genetic potential for resistance, transcriptomics reveals which genes are actively expressed (Domínguez et al., 2017). In this method, illumina RNA-seq remains the standard due to its accuracy and depth (McGettigan, 2013). However, long-read approaches, such as Nanopore, PacBio Iso-Seq etc., are increasingly applied to resolve operon structures and full-length transcripts (McGettigan, 2013; Zhao et al., 2019).

The primary output in transcriptomics is a genome-wide quantitative expression matrix indicating which genes are up- or downregulated (Domínguez et al., 2017). In the context of antibiotic resistance, such profiles can detect overexpression of efflux pumps, β-lactamase enzymes, porins and stress-response pathways that directly contribute to resistance phenotypes. For example, a clinical isolate may show marked overexpression of the *mexXY* efflux operon, suggesting active aminoglycoside efflux. Additionally, transcriptomics also captures adaptive and tolerance-related mechanisms, including induction of oxidative stress genes, biofilm pathways, and quorum-sensing regulators that enhance survival without requiring genetic mutations.

In one study, transcriptome profiling has been applied to understand *P. aeruginosa* resistance (Khaledi et al., 2016). RNA-seq of 135 clinical isolates was used in a transcriptome-wide association study to find gene expression patterns linked to resistance to fluoroquinolones, β-lactams, and aminoglycosides. They identified not only the usual suspects, such as, efflux genes or β-lactamase, but also potential novel resistance biomarkers with expression correlating to drug resistance. This suggests that transcriptomic analysis can improve accuracy of AST prediction. Another study provided a direct demonstration of transcriptomic complementation of genomic prediction was provided (Khaledi et al., 2020). The study integrated genomic and transcriptomic data from 414 clinical *P. aeruginosa* isolates. Their ML classifiers, when trained on genome sequence data alone, achieved sensitivity values of 0.8–0.9 for predicting resistance to tobramycin, ciprofloxacin, meropenem and ceftazidime. However, when gene expression profiles were incorporated alongside genomic data, predictive performance improved for all drugs except ciprofloxacin, with some combinations reaching sensitivity values above 0.9. This improvement was attributed to the ability of transcriptomic data to capture regulatory shifts that are invisible at the DNA sequence level.

Despite these advantages, clinically transcriptomics is not yet a routine diagnostic tool due to its high cost and requirement of even more complex analysis than WGS (Moore and Seibold, 2022). RNA-seq requires high-quality RNA extraction and deep sequencing, both of which are technically demanding and costly for routine clinical laboratories (Ozsolak and Milos, 2011). Moreover, expression can vary depending on growth phase or environment, and the expression profile *in vitro* might not reflect *in vivo* conditions. Additionally, RNA instability necessitates rapid processing (Ozsolak and Milos, 2011). These limitations, along with the lack of widely validated reference standards, currently constrain the clinical deployment of transcriptomics. Nonetheless, transcriptomics provides valuable functional insight into active resistance mechanisms, offering a crucial complement to genomic data.

### 5.4 Proteomics

Proteomics provides a direct, functional assessment of antimicrobial resistance in *P. aeruginosa* by identifying and quantifying proteins that mediate resistance phenotypes (Pérez-Llarena and Bou, 2016). Mass spectrometry (MS) is the central technology in clinical proteomics (Pérez-Llarena and Bou, 2016; Birhanu, 2023). The most widely implemented platform, MALDI-TOF (Matrix-Assisted Laser Desorption Ionization Time-of-Flight) MS, is routinely used for rapid species identification based on protein fingerprints and has recently been adapted for resistance detection (Vrioni et al., 2018). The MALDI Biotyper Detection System is a major advancement in this field (Sogawa et al., 2011). It combines MALDI-TOF spectral analysis with a curated reference database system. This platform enables automated detection of β-lactamase activity directly from bacterial colonies or positive blood cultures. By comparing pre- and post-incubation spectra, the Biotyper can confirm enzymatic hydrolysis of antibiotics such as carbapenems or cephalosporins. This provides clinically actionable results within a short timeframe. In one study, a resistant, carbapenemase-producing *P. aeruginosa* hydrolyzed imipenem, yielding mass peaks corresponding to breakdown products, whereas a susceptible strain did not (Miltgen et al., 2018). Beyond whole-cell profiling, proteomic analyses can be performed by shotgun LC-MS/MS, which digests and quantifies thousands of proteins, or by targeted MS methods designed to detect specific resistance-associated proteins (Wenk et al., 2024). Newer MALDI-TOF assays, such as MALDIxin, extend proteomic detection to lipid A modifications, particularly phosphoethanolamine or L-Ara4N additions which are characteristic of colistin resistance (Solntceva et al., 2021).

Proteomics offers several advantages as a diagnostic modality. Since it measures proteins directly, it confirms whether resistance determinants are present and active, thereby validating genomic predictions and distinguishing expressed resistance from silent genetic potential (Pérez-Llarena and Bou, 2016). MALDI-TOF MS provides extremely rapid turnaround times and is already widespread in clinical microbiology laboratories, facilitating integration of resistance assays with minimal added infrastructure (Solntceva et al., 2021; Birhanu, 2023). Additionally, proteomic approaches can detect unexpected biomarker patterns or post-translational modifications (PTMs) that are not identifiable through genomic analyses (Solntceva et al., 2021).

Nonetheless, proteomic detection is constrained by dynamic range limitations. Low-abundance proteins, such as some porins or regulatory enzymes, may be difficult to detect in whole-cell spectra (Chandramouli and Qian, 2009). Additionally, MALDI-TOF identification libraries have limited capacity to resolve subtle resistance determinants, often requiring specific hydrolysis assays or targeted workflows (Greco et al., 2018). Moreover, LC-MS/MS methods involve high cost, complex sample preparation and lower throughput compared to sequencing (Greco et al., 2018).

Overall, proteomics provides a rapid and functionally meaningful layer of resistance detection, particularly for enzymatic inactivation and efflux pump overexpression. It also serves as an important complement to genomic and transcriptomic diagnostics.

### 5.5 Lipidomics

Lipidomics offers critical insight into membrane associated resistance mechanisms in *P. aeruginosa* through a comprehensive molecular analysis of the lipid constituents (Han, 2016). Using LC-MS/MS, GC-MS or MALDI-TOF workflows, lipidomics quantifies lipid species and detects chemical modifications that alter membrane structure and charge, typically through a spectrum or chromatogram mapping (Griffiths and Wang, 2009; Jurowski et al., 2017). Because *P. aeruginosa* possesses a highly adaptable cell envelope, including lipid A, phospholipids and outer membrane glycolipids, lipidomic profiling is uniquely positioned to reveal membrane remodeling events that drive antibiotic resistance.

One of the most clinically important applications of lipidomics is the detection of lipid A modifications that cause polymyxin (colistin) resistance (Gerhardtova et al., 2024). In *P. aeruginosa*, activation of the PmrAB or PhoPQ regulatory systems leads to the addition of L-Ara4N or phosphoethanolamine (PEtN) to lipid A, reducing outer-membrane charge and decreasing colistin binding (Gerhardtova et al., 2024). Lipidomic assays can directly distinguish these modified lipid A species from their unmodified forms. The MALDIxin assay, a rapid MALDI-TOF method, identifies diagnostic peaks of L-Ara4N or PEtN-modified lipid A in under an hour, providing a mechanism-specific alternative to colistin MIC testing (Solntceva et al., 2021).

Beyond polymyxin resistance, lipidomics also can identify shifts in phospholipid composition, changes in fatty acid saturation or alterations in membrane rigidity (Hewelt-Belka et al., 2016). In a research study, mutation of a lipid metabolism regulator (PA3911) caused dramatic phospholipid imbalances and sensitized *P. aeruginosa* to multiple antibiotics (Groenewold et al., 2018). Additionally, lipidomics can provide insights into phenotypic adaptation by documenting changes in lipid metabolism under stress conditions that influence tolerance phenotypes (Dessenne et al., 2024; Solano et al., 2025). Such findings highlight how lipidomics can reveal mechanistic links between membrane biochemistry and antimicrobial susceptibility.

However, at present, lipidomics for *P. aeruginosa* AMR detection remains primarily a research tool, as no commercially validated lipidomic platforms for routine antimicrobial susceptibility testing currently exist. The strongest near-clinical rationale lies in polymyxin resistance detection, where the MALDIxin test correctly identified all susceptible and most resistant *P. aeruginosa* strains (Solntceva et al., 2021). In this application, lipidomics functionally complements genomics by confirming lipid A modifications (Jeannot et al., 2021). However, requirements for negative-ion mode-capable instrumentation and the absence of harmonized reference measurements and standardized lipid libraries remain critical translational barriers (Vvedenskaya et al., 2022; Becker and Lupetti, 2023). In addition, lipidomics faces notable limitations. Broad lipidomic profiling typically requires solvent extraction, specialized MS instrumentation and expert interpretation (Jurowski et al., 2017). Furthermore, lipidomics covers only a subset of resistance mechanisms and must be integrated with other omics approaches for full diagnostic coverage (Armitage and Southam, 2016). Nonetheless, lipidomics remains as a highly informative tool to identify membrane-mediated antibiotic resistance in *P. aeruginosa*.

### 5.6 Metabolomics

Metabolomics involves the analysis of chemical metabolites produced or used by organisms. In the context of diagnostics, metabolomic approaches seek to identify unique metabolic signatures of *P. aeruginosa* or detect changes in metabolites in response to antibiotics (Mielko et al., 2019). Using technologies such as LC-MS/MS, GC-MS, and NMR spectroscopy, metabolomics captures both intracellular and extracellular profiles of the metabolome (Griffiths and Wang, 2009; Munjal et al., 2022).

One application is using volatile organic compounds (VOCs) or other metabolites as biomarkers for infection (Monteiro et al., 2013; Sethi et al., 2013; Hong-Geller and Adikari, 2018; Sadaka et al., 2024). VOCs are organic chemical compounds produced by the bacterium during its metabolism that functions as signaling molecules and secondary metabolites. One distinct VOC produced by *P. aeruginosa* is 2-aminoacetophenone, which is a quorum-sensing molecule, detected in breath or sputum of infected patients of cystic fibrosis (Sethi et al., 2013; Mielko et al., 2019). The most advanced application is VOC-based detection of *P. aeruginosa* infection in cystic fibrosis patients using electronic nose devices and ion mobility spectrometry, which can identify infection from exhaled breath (Robroeks et al., 2010; Licht et al., 2023). Some pilot studies have demonstrated sensitivities above 80% for distinguishing *P. aeruginosa-infected* CF patients from non-infected controls (Sethi et al., 2013; Pabary et al., 2016). Another study developed a miniaturized VOC sensor platform that enables rapid, label-free antibiotic susceptibility testing by detecting VOC emission kinetics from bacterial metabolism, determining MICs for multiple antibiotics (Park et al., 2026). Depending on the bacterial strains, growth conditions and the environment, *P. aeruginosa* produces a wide range of VOCs. These include dimethyl sulfide (DMS), dimethyl disulfide (DMDS), 2,5-dimethylpyrazine (2,5-DMP), hydrogen cyanide (HCN), ketones, indoles, phenols and various hydrocarbons (Sethi et al., 2013).

Another metabolomic approach is to directly detect antibiotic degradation products or metabolic changes upon antibiotic exposure (Vincent et al., 2016). For example, if *P. aeruginosa* is incubated briefly with an antibiotic, resistant strains might metabolize or modify the drug, producing a distinctive metabolite profile. Mass spectrometry could potentially pick up these signals faster than waiting for visible growth. One study used LC-MS to detect the hydrolysis of β-lactams by resistant strains (Serafim et al., 2020). Similarly, metabolomics can support identification of phenotypic adaptation through the identification of biofilm-associated metabolites, quorum-sensing molecules, cyanide production or metabolic signatures of nutrient limitation (Vitale et al., 2024). Additionally, metabolomics may indirectly suggest efflux-related resistance through altered extracellular accumulation of certain metabolites or drugs (Thakur et al., 2025).

However, the application of metabolomics specifically for resistance detection is still in the proof-of-concept phase. One practical barrier of metabolomics is metabolome is highly dynamic and sensitive to environmental variation, which complicates standardization and reproducibility (Monteiro et al., 2013). Additionally, sample preparation often requires rapid specialized extraction procedures. Moreover, LC-MS and NMR instrumentation demand significant expertise (Emwas et al., 2013). Furthermore, metabolite-based resistance biomarkers are still under development and host-derived metabolites in clinical specimens may obscure bacterial signals (Monteiro et al., 2013; Aarika et al., 2025). As a result, in clinical practice, metabolomics-based diagnostics for *P. aeruginosa* remain at the translational stage. However, metabolomics can be best positioned as an adjunct to genomic and proteomic data within integrated multi-omics frameworks, where it can add a functional dimension by detecting the biochemical consequences of resistance mechanisms that other omics layers predict but cannot directly observe (Subanovic et al., 2025).

### 5.7 Phenomics

Phenomics refers to the systematic measurement of observable bacterial behaviors, such as, growth, survival, metabolism, stress responses etc., across diverse conditions, including exposure to antibiotics (Houle et al., 2010; Acin-Albiac et al., 2020). Phenomics directly and rapidly measures the functional outcome of antibiotic action (Acin-Albiac et al., 2020).

High-throughput culture-based systems such as VITEK 2, BD Phoenix M50, Microscan and the Accelerate Pheno™ platform use optical sensors, fluorescence imaging or microfluidic confinement to monitor growth kinetics under antibiotic exposure (Marschal et al., 2017; Bergquist, 2022; Ghavami et al., 2025; Majumder et al., 2025). Accelerate Pheno immobilizes *P. aeruginosa* cells from positive blood cultures in a microfluidic cartridge and uses automated microscopy to quantify replication in each antibiotic condition, yielding MICs within 7 h (Marschal et al., 2017; Fernandez-Pittol et al., 2019). This system is already FDA-cleared and commercially deployed in hospital laboratories (Pancholi et al., 2018). Clinical evaluations have demonstrated categorical agreement rates of 93–97% with standard broth microdilution for *P. aeruginosa* (Charnot-Katsikas et al., 2018; Hattab et al., 2024). Similarly, the VITEK 2 system uses turbidimetric growth detection in microwell cards to deliver automated MIC results in 4–18 h from isolated colonies, with categorical agreement of 90–96% against broth microdilution for Gram-negative bacilli including *P. aeruginosa* (Joyanes et al., 2001; Eigner et al., 2005; Bobenchik et al., 2017; Khan et al., 2021). On the other hand, QuickMIC utilizes a stable antibiotic gradient inside a microchannel and tracks where bacterial growth ceases, enabling MIC determination in 3–4 h (Bergquist, 2022; García-Rivera et al., 2024). A growth-independent approach using ML-assisted nanomotion technology, which detects nanoscale bacterial vibrations via cantilever sensors, achieved 89.5–98.9% accuracy for cephalosporin and fluoroquinolone susceptibility in *E. col*i and *K. pneumoniae* from positive blood cultures within 2–4 h (Sturm et al., 2024). The BD Phoenix system achieves comparable performance within 6–16 h, with categorical agreement of 93–97% (Eigner et al., 2005; Duggal et al., 2012; Hong et al., 2019; Tsai et al., 2021). Another form of phenomics includes Phenotype MicroArrays (Biolog), which measure respiration or growth across about 2,000 environmental and antibiotic conditions (Bochner et al., 2001). These assays generate metabolic and physiological fingerprints that can uncover collateral sensitivities, metabolic vulnerabilities or efflux-related phenotypes. For example, Biolog dye-reduction patterns change when efflux pumps are inhibited or when resistant mutants acquire metabolic defects. Such platforms reveal emergent properties of the cell and offer insights into resistance-associated physiology, although they are slower and less focused than rapid AST systems.

A major innovation in phenomics is the rise of single-cell functional assays. Techniques like single-cell Raman microspectroscopy with D_2_O labeling quantify metabolic activity at the level of individual bacterial cells (Ho et al., 2019). Because metabolic incorporation of deuterium produces characteristic carbon-deuterium (C-D) Raman signatures, antibiotic susceptibility can be determined by whether cells continue synthesizing macromolecules under drug pressure. Resistant cells exhibit strong C-D signals, while susceptible cells show metabolic arrest. Studies have shown this method can distinguish resistant from susceptible *P. aeruginosa* within 2–3 h (Liu et al., 2023). Microfluidic single cell arrays similarly isolate individual bacteria to monitor immediate growth responses. Thus, it can detect heteroresistance or persister subpopulations that bulk AST may miss (Lu et al., 2023). These single-cell approaches are especially powerful for characterizing phenotypic adaptation, including tolerance and transient survival strategies.

However, limitations in phenomics include dependence on viable culture, cost of advanced instruments, variability influenced by growth conditions, and inability to directly identify the molecular basis of resistance (Ying, 2023). Some resistant subpopulations may escape detection if they grow too slowly within the assay window. Overall, phenomics provides a ground-truth functional measurement of antimicrobial susceptibility.

**FIGURE 2:**
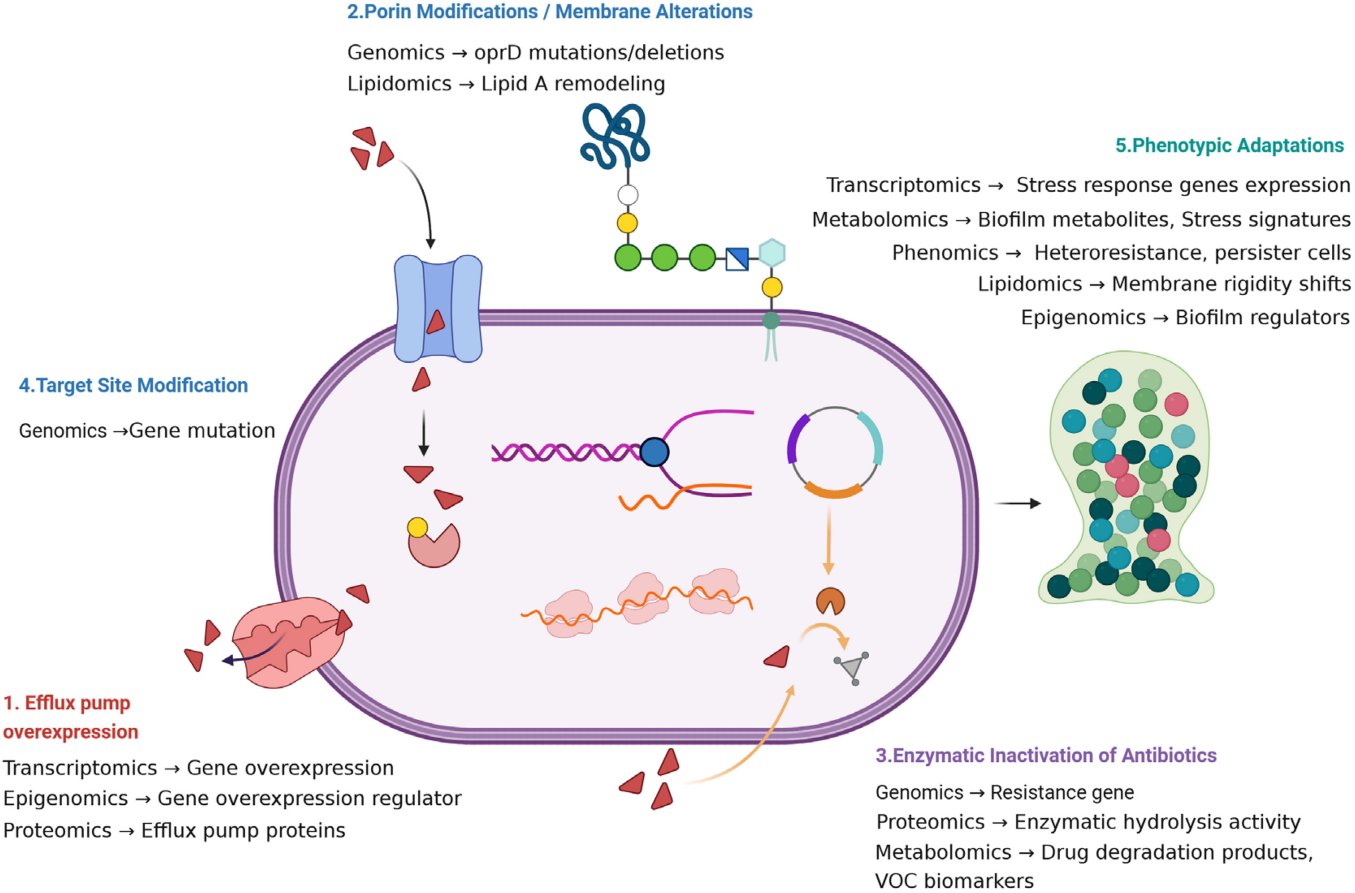
Mapping of antimicrobial resistance mechanisms in *P. aeruginosa* to omics-based diagnostic platforms. Each of the five major resistance strategies- efflux pump overexpression, porin and membrane changes, enzymatic antibiotic inactivation, target site mutations and phenotypic adaptations such as biofilm and persister cells is detectable by a distinct set of omics tools. Created in https://BioRender.com.

In summary, omics-based diagnostics provide complementary insights into the complex resistance strategies of *P. aeruginosa*. Genomics defines the resistome, epigenomics reveals regulatory methylation patterns, transcriptomics identifies actively expressed resistance pathways, proteomics detects functional resistance enzymes, lipidomics uncovers membrane modifications such as lipid A remodeling, metabolomics captures chemical signatures of stress or drug degradation and phenomics provides the definitive functional readout of susceptibility. The relationship between each resistance mechanism and the omics platforms best suited to detect it is illustrated in Figure 2. Individually, each omics platform illuminates a distinct facet of resistance, yet each also carries inherent blind spots, such as, genomics cannot confirm gene expression, transcriptomics cannot verify protein production and phenomics cannot identify the molecular mechanism. Only by integrating these complementary layers into a unified multi-omics framework, as discussed in the following section, can the full complexity of *P. aeruginosa* resistance be resolved for clinical decision-making.

## 6 Multi-omics integration for precision therapeutics

**FIGURE 3:**
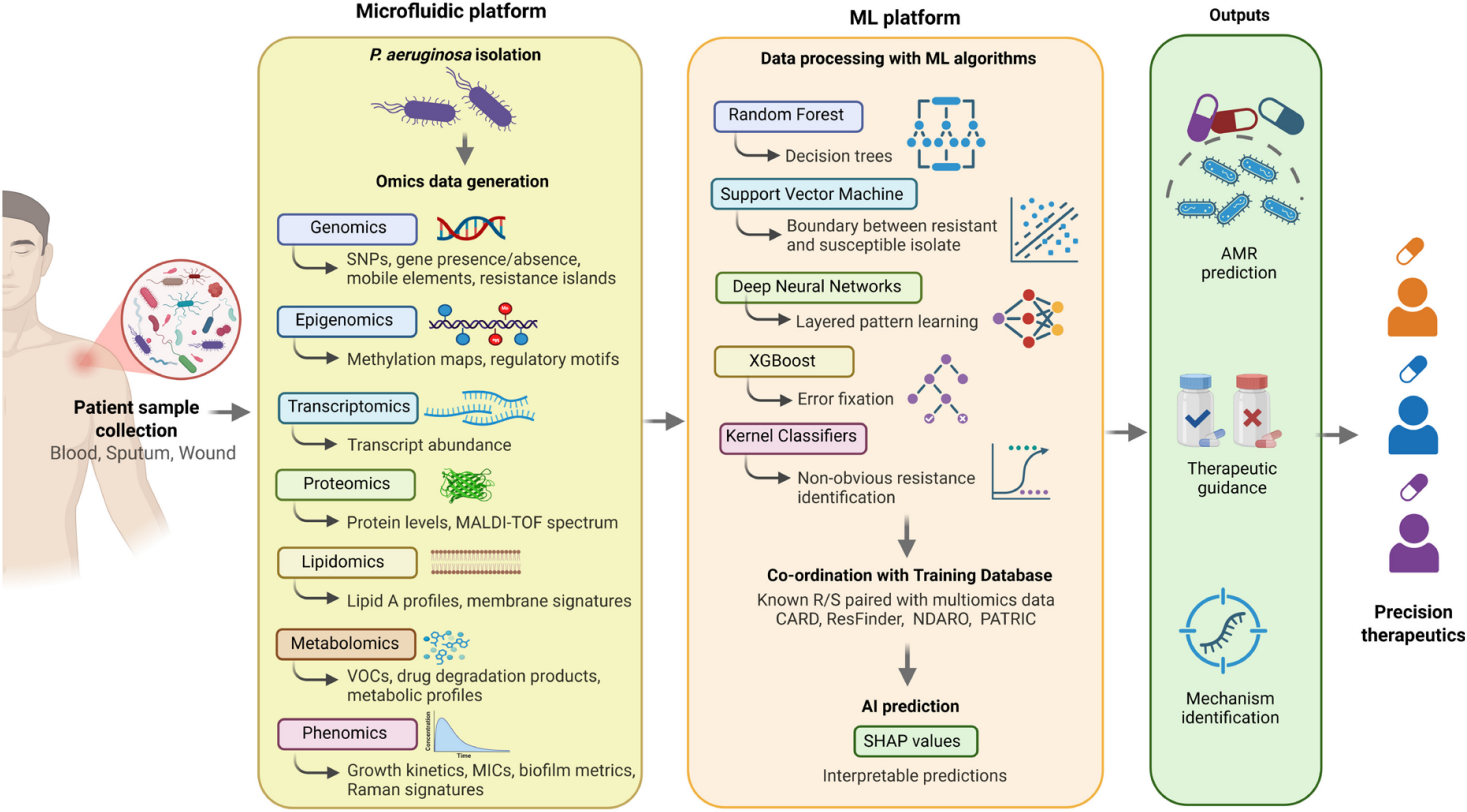
Integrated framework of the proposed multi-omics and machine learning pipeline for *P. aeruginosa* diagnostics. Starting from a patient sample, the pipeline moves through three stages- bacterial isolation and parallel omics data generation on a microfluidic platform, processing of the combined molecular data using multiple ML algorithms coordinated with established resistance databases, and delivery of clinically actionable outputs including AMR predictions, resistance mechanism identification and therapeutic guidance. The pipeline is a proposed conceptual framework and has not yet been validated as an integrated clinical system. Created in https://BioRender.com.

Because antimicrobial resistance in *P. aeruginosa* arises from intertwined genetic, regulatory, metabolic and phenotypic processes, no single omics platform can fully capture the complexity of its adaptive landscape (Pei et al., 2025). Multi-omics integration refers to the combined analysis of genomic, epigenomic, transcriptomic, proteomic, lipidomic, metabolomic and phenomic data (Subramanian et al., 2020). This process offers a systems-level framework capable of linking molecular determinants to functional outcomes. By correlating genotype with phenotype, integrated omics can resolve ambiguities that arise when individual datasets are interpreted in isolation (Babu and Snyder, 2023). A schematic overview of this integrated multi-omics diagnostic framework is shown in Figure 3, illustrating how multiple omics layers converge to improve resistance detection and prediction.

A typical example involves β-lactam resistance mediated by *ampC*. WGS may detect an *ampC* allele, however, this alone does not establish whether resistance is phenotypically expressed. But by integrating transcriptomic data, it can be confirmed whether the *ampC* gene is overexpressed or not. If *ampC* mRNA levels are extremely high, this supports active AmpC-mediated resistance. In contrast, if expression is low, *ampC* gene maybe present but not strongly expressed, and the strain might be susceptible unless induced. Similarly, proteomic data from MALDI-TOF can confirm the production of specific β-lactamase peptides, while metabolomic data may reveal the functional consequences, such as detection of antibiotic degradation products in the culture media. By layering these insights, clinicians can get a holistic view of the pathogen. This is particularly valuable for *P. aeruginosa*, where resistance can be multi-factorial.

Systems-level integration builds on these relationships by connecting transcriptional programs to metabolic fluxes and downstream physiological outcomes. For instance, integrating RNA-Seq and metabolomics within a genome-scale metabolic model has shown how polymyxin-rifampicin combination therapy disrupts central carbon metabolism, lipid biosynthesis and peptidoglycan pathways, revealing coordinated stress responses not visible from single-omic analysis (Mahamad Maifiah et al., 2022). Similarly, overlaying transcriptomic upregulation of efflux pumps onto metabolomic signatures of altered toxin accumulation enables mechanistic inference about efflux-driven resistance (Blanco et al., 2018). These integrative approaches often uncover tolerance and adaptation pathways, such as, quorum-sensing suppression, metabolic arrest or lipid remodeling.

In practice, multi-omics integration for diagnostics is still in development. However, ML increasingly serves as the analytical engine enabling multi-omics integration. Some ML models are emerging that analyze genomic and transcriptomic data to predict antibiotic MICs against bacterial strains (Sastry et al., 2021; Bansal et al., 2023). ML algorithms like random forests, deep neural networks, and kernel-based classifiers are trained on multi-omics data from known resistant and susceptible bacterial isolates (Bansal et al., 2023). By learning the complex genotypic and phenotypic relationships, the model can identify the rules that distinguish resistant from susceptible strains. In one study, random-forest models built on presence or abundance genes achieved AUC > 0.8 for predicting resistance to imipenem, meropenem, piperacillin-tazobactam and levofloxacin in *P. aeruginosa* (Cao et al., 2024). Another study applied random forest and BioWeka classifiers to WGS data from *P. aeruginosa* isolates, reporting mean classification accuracy of ≥ 96–98% across 12 antibiotic classes (Noman et al., 2023). An mNGS-based ML model for *Acinetobacter baumannii* identified AMR signatures from 1,942 genomes and classified resistance to imipenem, ceftazidime, cefepime and ciprofloxacin with accuracies of more than 97% (Hu et al., 2023). Another research group developed an explainable two-tier ML pipeline using k-mer features from *P. aeruginosa* genome sequences and achieved an AUROC value of 0.77 with 84% sensitivity (Jain et al., 2025). Moreover, ML is particularly effective at identifying complex genetic relationships involving multiple genes or subtle interactions that are difficult to find with other methods. One study built an algorithm combining *P. aeruginosa* genome sequences and transcriptomes to predict ceftazidime and meropenem susceptibility, finding it more reliable than using either data type alone (Khaledi et al., 2020). Building on this transcriptomic approach, another study used a genetic algorithm to identify minimal gene signatures (35–40 genes) from 414 clinical *P. aeruginosa* isolates and achieved 96–99% accuracy (F1 scores 0.93–0.99) for predicting resistance to meropenem, ciprofloxacin, tobramycin and ceftazidime (Shahreen et al., 2025). One research group also demonstrated that ML models trained on MALDI-TOF, Raman spectroscopy, whole-genome sequencing and microcalorimetry data could predict tobramycin susceptibility of *P. aeruginosa* biofilms (Vergauwe et al., 2025). They achieved 97.8% accuracy for planktonic MIC prediction and 80.4% for biofilm prevention prediction. These studies collectively demonstrate that ML-based AMR prediction for *P. aeruginosa* is approaching clinically useful performance levels across multiple data modalities.

Recent advances have expanded this field significantly. Deep learning models trained on large MALDI-TOF spectral datasets now predict resistance directly from proteomic fingerprints, while explainable AI tools help identify which spectral features correspond to resistance determinants (Weis et al., 2022). In one study, the authors trained AI models on clinical MALDI-TOF mass spectra to predict resistance to ceftazidime/avibactam and ceftolozane/tazobactam, and achieved AUROC values of 0.869 and 0.856, respectively, with specificity above 0.89 (Nguyen et al., 2024). A large multi-site study using over 300,000 MALDI-TOF spectra achieved AUROCs of 0.80–0.74 across a number of pathogens and using VITEK^®^ MS-derived spectra from 2,229 clinical isolates, CatBoost achieved an AUROC of 0.91 (Weis et al., 2022; López-Cortés et al., 2025). In addition, new sequence-based deep learning methods, including chaos game representation of genomes and protein language models can predict antimicrobial resistance from genomic architecture or protein sequence alone, which strengthen the genomic and proteomic layers of multi-omics diagnostics (Löchel et al., 2020; Luo and Cai, 2025). Additionally, bioinformatic platforms such as DeepARG, Resistomap, PathoFact, and similar platforms demonstrate how ML-driven pipelines can classify resistance determinants, quantify ARG burdens, and identify mobile genetic elements at scale (Arango-Argoty et al., 2018; Gupta et al., 2020; De Nies et al., 2021; Madrigal et al., 2022).

Integrating multi-omics with microfluidic platforms would be a revolutionary step that offers the speed and automation necessary for clinical diagnostics (Khaledi et al., 2020). In this scenario, a fully integrated platform could isolate *P. aeruginosa* from a patient sample, perform rapid WGS and targeted RNA profiling, monitor lipid A modifications through MALDI-TOF, and simultaneously record metabolic responses to antibiotics, all within a miniaturized device. Feeding these multi-layered data into ML models could yield near-real-time predictions of drug susceptibility, surpassing the speed and resolution of conventional AST. Although technically demanding, such microfluidic multi-omics platforms represent a plausible trajectory toward point-of-care precision microbiology.

The approximate timings of the integrated diagnostic workflow can be broken down stepwise, as illustrated in Figure 1. In the first phase, microfluidic platforms would receive the clinical specimen and perform on-chip sample preparation within 1–2 h (Zhang et al., 2020; Kaprou et al., 2021; Aldea et al., 2025). This step would include bacterial capture, cell lysis, and molecular extraction, all within miniaturized nano- to picoliter reaction volumes that would accelerate diffusion and binding kinetics far beyond conventional methods. In the second phase, single-cell or droplet-based microfluidic AST could detect bacterial growth or metabolic changes, with label-free systems capable of delivering MIC or S/R calls within 2–4 h (Zhang et al., 2020; Kaprou et al., 2021; Abubakar et al., 2025; Aldea et al., 2025). Concurrently, parallel multi-omics modules could proceed alongside microfluidic phenotyping. Among these, nanopore-based metagenomics has been shown to identify species and resistance genes through real-time analysis within 2–7 h of sequencing, MALDI-TOF mass spectrometry can deliver proteomic identification in minutes per isolate, and targeted transcriptomic or metabolomic profiling has been demonstrated within 2–4 h (Athamanolap et al., 2019; Bhattacharyya et al., 2019; Charalampous et al., 2019; Kaprou et al., 2021; Cheng et al., 2022; Weis et al., 2022; Ahmad et al., 2023; Gao et al., 2024; Sauerborn et al., 2024; Sturm et al., 2024; Aldea et al., 2025; López-Cortés et al., 2025). In an idealized configuration, these omics modules could run simultaneously alongside microfluidic AST, with the total turnaround dominated by the longest single module. However, in practice, different omics layers require fundamentally distinct sample preparation conditions. Parallelizing these divergent preparation workflows within a single microfluidic cartridge remains a major unresolved engineering challenge (Cunha et al., 2022; Abubakar et al., 2025). In the final phase, trained ML models could convert the accumulated multi-omics features into antibiotic-specific susceptibility predictions within seconds to minutes, with SHAP-based explainable AI capable of identifying the molecular drivers behind each call (Khaledi et al., 2020; Zhang et al., 2020; Wang et al., 2022; Hu et al., 2023; Zagajewski et al., 2023; Sturm et al., 2024). However, automated quality control checks and report generation may add up to an additional hour to the pipeline. Thus, multi-omics modules integrated on a microfluidic platform with ML-driven analysis could substantially compress diagnostic turnaround relative to the 48–72 h required by conventional culture-based AST. A comprehensive multi-omics workflow could potentially be completed within 8–12 h. Therefore, integrated approach would represent a substantial improvement, potentially achievable within a single clinical workday and would provide mechanistic depth far exceeding conventional AST.

Despite its promise, multi-omics integration faces significant challenges. Data heterogeneity, differences in scale and noise and the requirement for paired datasets complicate analysis (Wang, 2017; Jan et al., 2025). Significant limitations must be acknowledged as well before ML models can be responsibly deployed in clinical settings. First, dataset bias is a pervasive concern. Most training datasets are derived from isolates collected in high-income countries and tertiary hospitals. Therefore, models on these datasets may perform poorly when applied to strains from underrepresented geographic regions (Cross et al., 2024; de la Lastra et al., 2024). Second, the black box nature of deep learning models limits clinical interpretability. A clinician cannot easily verify why a model predicts resistance, which undermines trust and complicates regulatory approval (Ito et al., 2025). However, Explainable AI (XAI) approaches, such as SHAP (SHapley Additive exPlanations) values and attention-based architectures, are beginning to address this gap by identifying which genomic or spectral features drive predictions (Tharmakulasingam et al., 2022; Praveen et al., 2024; Forkuo et al., 2025; Warrier et al., 2026). Third, temporal drift poses a challenge in *P. aeruginosa* as its resistance mechanisms evolve continuously. ML models trained on historical data may lose accuracy as new mutations and mobile genetic elements emerge (Bilal et al., 2025; Ito et al., 2025).

Beyond these analytical challenges, multi-omics diagnostics face substantial practical barriers to clinical adoption. Cost remains a primary concern, as a single multi-omics analysis combining WGS, RNA-Seq and mass spectrometry-based proteomics for one isolate can exceed $1,000 depending on platform and context, compared with $15–30 for conventional disk diffusion AST (Marshall et al., 2022; Price et al., 2023; Tran et al., 2023). Although costs are declining, the aggregate expense of multi-layered omics profiling remains a challenge for routine use in most healthcare settings. However, the cost-effectiveness of rapid diagnostics may be view as context-dependent. A systematic review demonstrated that rapid molecular identification combined with antimicrobial stewardship reduced bloodstream infection mortality by up to 23% and saved an average of $4,140 per case through shorter ICU stays and earlier targeted therapy (Raycheva et al., 2026). These savings are most pronounced in critically ill patients, where each additional ICU day costs around $3,000 (Halpern and Pastores, 2010). For less severe infections, however, the incremental benefit over conventional AST may not justify the higher cost. A stratified diagnostic model, as discussed in section 7.5, could address this cost-benefit asymmetry by matching diagnostic depth to clinical severity. Additionally, no comprehensive, integrated multi-omics diagnostic platform for AMR has yet received regulatory approval from the FDA or achieved CE-IVD status for routine clinical use. While individual omics technologies are making strides, integrated multi-omics diagnostic platforms remain largely in research, development, or Laboratory Developed Test (LDT) phases. Additionally, computational intensity and the difficulty of experimentally validating systems-level predictions also limit widespread clinical adoption. These challenges highlight that while multi-omics integration offers unmatched diagnostic depth, its translation from research to bedside will require deliberate investments in cost reduction, workflow automation, regulatory frameworks, and clinical interpretive standards.

## 7 Future perspectives

Overcoming the barriers in adopting multi-omics diagnostic procedure in clinical settings will require a coordinated roadmap spanning clinical validation, standardization, infrastructure development, economic planning and workforce training. Below, we outline the key steps necessary to translate multi-omics diagnostics from proof-of-concept research into routine clinical practice for antibiotic-resistant *P. aeruginosa*.

### 7.1 Implementation roadmap

A phased adoption model is the most realistic pathway for clinical integration. In the near term, clinicians can integrate commercially available rapid phenomic platforms, such as Accelerate Pheno™ and VITEK 2, alongside existing MALDI-TOF species identification to achieve faster AST without major infrastructure overhaul. Next, hospitals with existing molecular diagnostics capacity can incorporate targeted genomic panels, such as nanopore-based resistome screening, alongside phenomic AST. This will create a two-layer diagnostic approach that combines mechanistic insight with functional susceptibility data. In the long term, fully integrated microfluidic multi-omics platforms, performing WGS, targeted transcriptomics, proteomic profiling and phenotypic AST within a single closed cartridge may enable point-of-care precision diagnostics. Early adopters are likely to be tertiary care hospitals managing high burdens of MDR infections, with broader adoption expanding as costs decrease and workflows are optimized (Committee on the Review of Omics-Based Tests for Predicting Patient Outcomes in Clinical Trials et al., 2012). Over time, as these technologies become more affordable and procedures are optimized, their adoption may extend to a broader range of healthcare settings.

### 7.2 Clinical adoption by physicians

A critical and often overlooked dimension of implementation is how clinicians will actually interact with and act upon multi-omics diagnostic outputs. Clinicians are not expected to interpret raw omics data; rather, results must be translated into simple, actionable reports. The reports should indicate predicted susceptibility, resistance mechanism, and confidence level integrated directly into electronic medical records (EMRs) and antibiotic stewardship decision-support tools (Litvin et al., 2012). Such clinical decision support systems (CDSS) have already demonstrated efficacy in improving antibiotic prescribing when linked to rapid diagnostic results (Holstiege et al., 2014). For multi-omics to succeed clinically, diagnostic laboratories must establish clear reporting standards that communicate genomic and phenotypic resistance predictions in formats familiar to infectious disease physicians and pharmacists. Furthermore, prospective clinical validation studies are urgently needed to evaluate whether multi-omics-guided antibiotic selection improves patient outcomes compared with conventional AST. These studies should include mortality rate, ICU length of stay, antibiotic de-escalation rates and re-infection rates among the patients. Such trials should prioritize high-risk populations where MDR *P. aeruginosa* is most consequential, including VAP, bloodstream infections and cystic fibrosis exacerbations.

### 7.3 Standardization and protocols

A major obstacle to clinical deployment of omics-based diagnostics is the absence of harmonized methodologies (D’Adamo et al., 2021; Mohr et al., 2024). For WGS-based antimicrobial susceptibility prediction, standardized criteria must be established for sequencing depth, read quality, assembly metrics and variant-calling pipelines. Similarly, consensus must emerge regarding which resistance gene databases (e.g., CARD, ResFinder, NDARO) and which ML tools should be used for clinical interpretation. In addition to that, regulatory bodies like Clinical & Laboratory Standards Institute (CLSI) and European Committee on Antimicrobial Susceptibility Testing (EUCAST) will need to provide guidance on using molecular signatures in AST reporting. This includes establishing evidence-based genotype-phenotype interpretive criteria and defining how to handle ambiguous or intermediate predictions. Similarly, for rapid phenotypic methods, such as microfluidic AST, breakpoints need to be adapted for shortened periods. Multi-omics data integration will additionally require standardized, potentially FDA-approved analytical pipelines that convert raw data into clinically interpretable outputs.

### 7.4 Data infrastructure

Omics-based diagnostics generate large, heterogeneous datasets requiring robust computational infrastructure (Wang et al., 2019; Tang, 2024). Healthcare systems will need secure, scalable data storage compliant with international privacy standards. Cloud-based or hybrid computing architectures may be necessary to support real-time analysis of multi-omics data. In addition, hospital systems must adopt FAIR data principles (Findable, Accessible, Interoperable, Reusable) (Lappan et al., 2024). This will allow genomic and phenotypic data to be integrated with electronic medical records, surveillance networks and regional AMR monitoring systems. Continuous updating and curation of ARG databases, mutation catalogs and strain-specific genomic signatures will be required to maintain diagnostic accuracy as *P. aeruginosa* continues to evolve.

### 7.5 Cost and accessibility

While the integrated multi-omics diagnostics seems expensive, costs of many technologies are trending down. Nanopore sequencing, benchtop mass spectrometry and microfluidic AST platforms are becoming significantly more affordable. From a health economics perspective, preventing prolonged ICU stays or ventilator dependence through timely targeted therapy can justify these investments (Kumar et al., 2006). To ensure equity, global initiatives may be required to fund these technologies in low-resource settings, where MDR *P. aeruginosa* poses a significant burden. Regional reference laboratories could provide centralized sequencing and analysis services for hospitals lacking local infrastructure. Additionally, rather than employing multi-omics as a universal replacement for conventional AST, a stratified, three-tiered diagnostic framework matched to clinical severity may offers a more pragmatic path. At Level 1 (Routine), the majority of *P. aeruginosa* infections, including uncomplicated urinary tract infections, wound infections, and community-acquired cases would be managed with established rapid phenomic platforms such as VITEK 2 or BD Phoenix. This will cost around $15–30 per test, requiring no additional capital investment beyond existing infrastructure (Stokes et al., 2020). At Level 2 (Targeted), high-risk patients, such as those with bacteremia, ICU-acquired infections, or known MDR colonization would additionally receive nanopore-based resistome screening which will cost $40–100 per sample (Ali et al., 2024; Liu et al., 2024; Osei Sekyere, 2025). At Level 3 (Comprehensive), full multi-omics data with ML interpretation ($500–1,000 per analysis) would be reserved for treatment failure, XDR/PDR infections, and chronic *P. aeruginosa* in cystic fibrosis, where the higher cost is justified by the disproportionate mortality and downstream expenditure (Price et al., 2023; Nourazarain and Vaziri, 2025). To ensure global equity, regional reference laboratories could centralize Level 2 and Level 3 services for hospitals lacking local infrastructure, with multiplexed sequencing and cloud-based ML analysis further reducing per-sample costs as throughput scales.

### 7.6 Workforce training

The adoption of omics diagnostics will require a trained multidisciplinary workforce. Clinical microbiologists will need training in microbial genomics, bioinformatics pipelines and ML interpretation frameworks. Likewise, technicians may need to learn to operate new devices like nanopore sequencers or microfluidic chips. In addition, physicians and pharmacists must be educated on how to interpret and apply omics-based diagnostic results in clinical decision making. Academic programs in clinical genomics, computational microbiology and diagnostic data science will play a critical role in sustaining this workforce.

### 7.7 Research and development priorities

Quality research and development is the most important part of the integrated omics diagnostics.

Specifically, three research priorities merit immediate attention. First, the development of automated, miniaturized multi-omics platforms, which will integrate sample preparation, sequencing, mass spectrometry and ML-based interpretation into a single closed cartridge. This represents the most critical engineering challenge. Recent advances in microfluidic integration with nanopore sequencing and on-chip MALDI-TOF suggest this goal is technically achievable within the next decade (Postek et al., 2022; Wu and Mu, 2024). Second, developing open-access, continuously curated multi-omics resistance databases specifically for *P. aeruginosa*. This dataset must link genomic variants, expression profiles, proteomic signatures, metabolomic fingerprints, lipidomic profiles, epigenomic modifications and phenotypic outcomes. These are essential for training next-generation ML diagnostic models, as current databases such as CARD and ResFinder remain genomics-centric. Third, priority research areas should include biofilm diagnostics, host response biomarkers, AI-driven predictive analytics and host-based therapy guidance through identification of biomarkers in *P. aeruginosa* infection.

In summary, overcoming antibiotic-resistant *P. aeruginosa* will require diagnostics that are not only rapid and accurate but also mechanistically informative and clinically actionable. As technologies become more accessible and AI-driven analytic pipelines mature, integrated multi-omics platforms hold the potential to transform antibiotic therapy selection from empiric decision-making to individualized, precision-guided intervention, ultimately reducing the global burden of MDR *P. aeruginosa* infections.

## 8 Conclusion

Antibiotic resistant *P. aeruginosa* remains one of the most challenging nosocomial pathogens globally, and classified as a high-priority pathogen in the 2024 WHO Bacterial Priority Pathogens List. Its resistance arsenal, spanning efflux pump overexpression, porin loss, enzymatic inactivation, target site modifications and phenotypic adaptations creates a multifactorial challenge that no single diagnostic approach can fully resolve. Conventional culture-based AST still remains as the clinical gold standard. However, this procedure requires 48–72 h, frequently fails to detect heteroresistance, biofilm-mediated tolerance, and does not provide any idea of the molecular mechanisms driving resistance. Therefore, clinicians rely on empiric broad-spectrum therapy during a critical therapeutic window.

This review has presented a comprehensive framework for addressing these limitations through the integration of seven omics-based diagnostic platforms- genomics, epigenomics, transcriptomics, proteomics, lipidomics, metabolomics, and phenomics. Each resolves distinct layers of the *P. aeruginosa* resistome. Genomics provides the genetic blueprint through resistome profiling via WGS, but cannot confirm whether resistance genes are functionally active. Transcriptomics and epigenomics bridge this gap by capturing gene expression dynamics and regulatory methylation patterns. Proteomics confirms the production of resistance enzymes such as AmpC β-lactamases and detects porin loss at the protein level. Lipidomics directly identifies lipid A modifications responsible for polymyxin resistance. Metabolomics detects the biochemical consequences of resistance, including antibiotic degradation products and biofilm-associated metabolites. Phenomics, the most clinically advanced layer, provides the functional ground truth of susceptibility through rapid AST platforms already deployed in hospital laboratories. The mechanism-to-omics mapping framework presented here demonstrates that integrating these complementary layers is essential for comprehensive resistance profiling. ML-based multi-omics integration represents a transformative frontier in *P. aeruginosa* AMR diagnostics. Random forest, deep learning, and explainable AI models trained on genomic, transcriptomic and proteomic data have demonstrated clinically promising predictive performance, with some achieving AUROCs exceeding 0.85 across multiple antibiotic classes. Platforms such as DeepARG and PathoFact further enable scalable resistance classification at the pipeline level. Looking ahead, integration of multi-omics with microfluidic technologies could compress diagnostic turnaround from days to potentially within a single clinical workday, fundamentally accelerating clinical decision-making. However, translating this potential into clinical practice requires action across several priority areas, such as large-scale trials demonstrating that multi-omics-guided antibiotic selection improves patient outcomes, standardization and regulatory approval, cost reduction and accessibility, database expansion, workforce training and finally clinical adoption by physicians.
